# Supplementary material for: Demonstration of End-to-End Automation of DNA Data Storage
Source: Sci Rep. 2019 Mar 21;9:4998. doi: 10.1038/s41598-019-41228-8 (PMC6428863; doi:10.1038/s41598-019-41228-8)
Supplement: Supplementary file 1 — Supplemental: Demonstration of End-to-End Automation of DNA Data Storage [file 41598_2019_41228_MOESM1_ESM.pdf]

Supplemental: Demonstration of End-to-End Automation of DNA Data Storage

Christopher N. Takahashi[1], Bichlien H. Nguyen[1,2], Karin Strauss[1,2], and Luis Ceze[1]

[1] *School of Computer Science and Engineering, University of Washington*
[2] *Microsoft Research*

September 2018

1 Multi-read, Multi-write

Fig. S1 Illustrates a method for temporarily separating the synthesis product eliminating the waste of material and enabling multiple reads to be done from the same DNA pool. Since the ONT MinION flow cells can be reused for up to 48 hours at room temperature or weeks under refrigeration and additional sequencing mastermix may be included in the syringe, this is the only modification necessary. Additionally the same system can be exploited to allow for additional writes that may be combined in the secondary storage vessel.

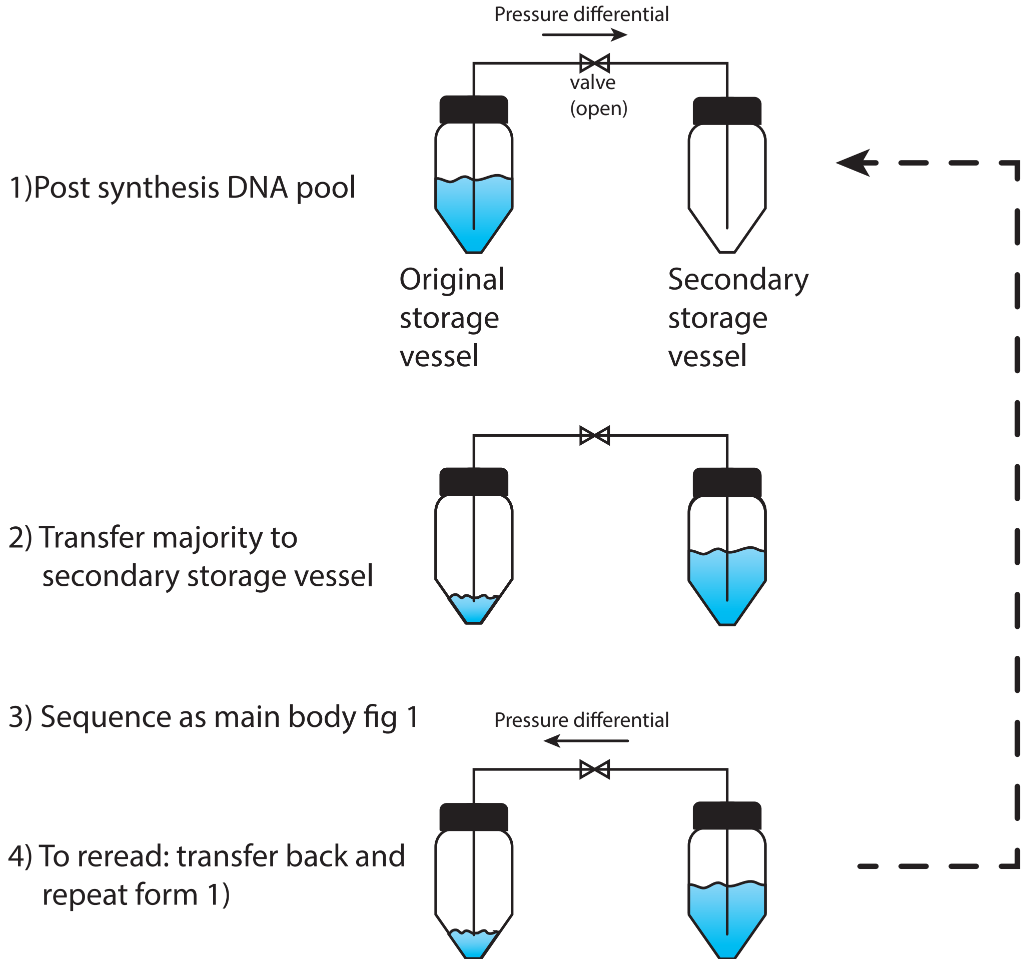

Figure S1: A proposed multi-read scheme. By the addition of two valves, one transfer, one for air(not shown) and a secondary storage vessel our system can be expanded to store excess synthesis material rather than discarding it. Additionally multiple synthesis products can be pooled together in the secondary storage vessel and read back at once.

2 Electrical Hookup

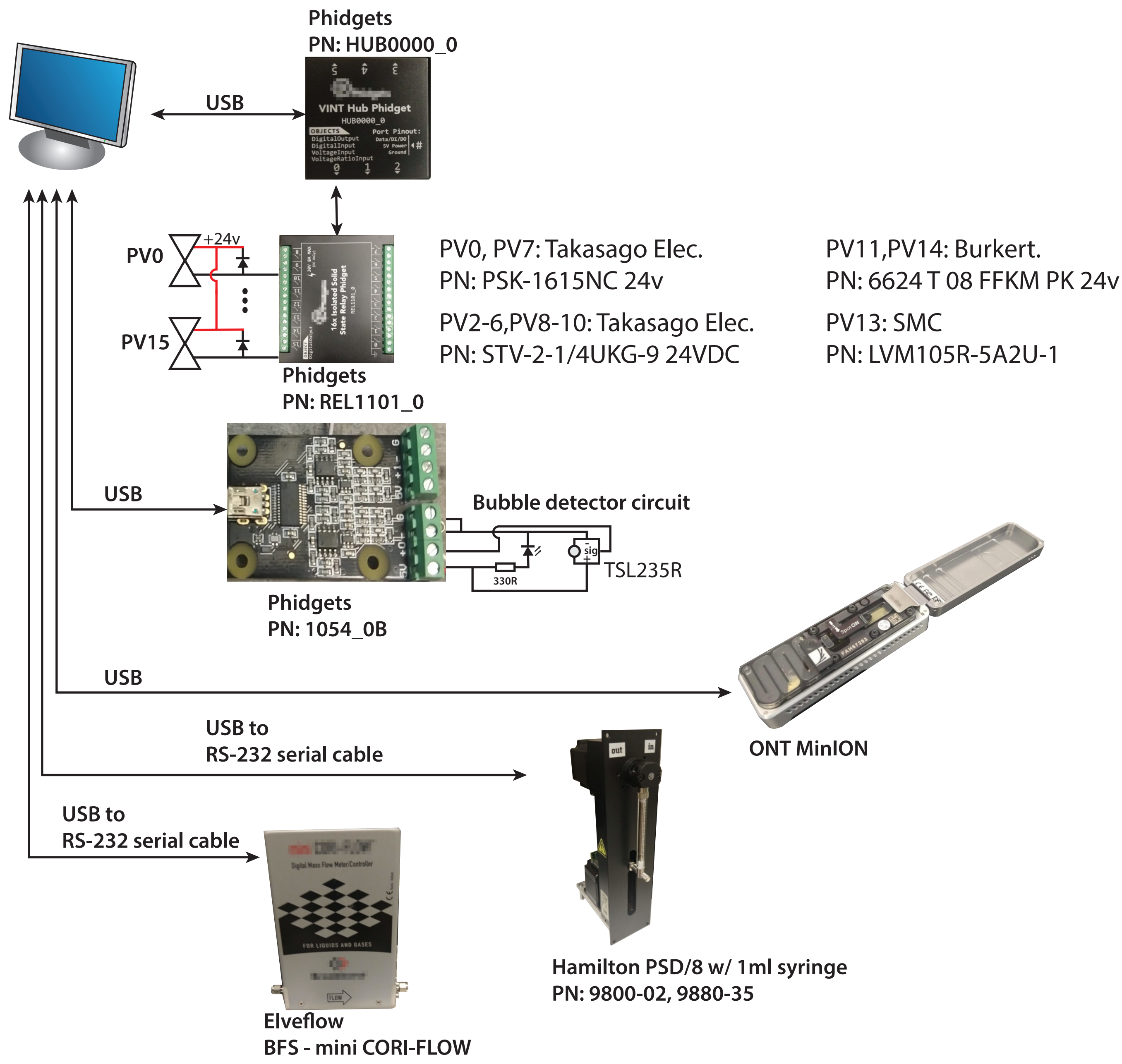

Figure S2: Electrical configuration for the end to end system. The majority of the parts consist of commercially available components with just a simple custom circuit for bubble detection and additional flyback diodes for the pinchvalves. 24 volts is provided to the pinchvalves by an external 2A at 24v wall adapter(not shown) with ground in common with the `REL1101_0` module.

3 Code

Hardware driving code is available at: `https://github.com/uwmisl/end2endController`

3.1 Hamming code/decode module

The python code implementing our hamming code/decode class is as follows:

3.1.1 HammingCode.py

```
#!/bin/env python2
import numpy as np
from numpy import dot
from hashlib import sha256

def _base4(x):
  return [x>>6&3,x>>4&3,x>>2&3,x&3]

def _ispowerof2(x):
  if x == 0:
    return false
  return (x & (x-1)) == 0

def _generate_HCMatrix(parody_bits):
  totalbits = 2**parody_bits-1
  databits = totalbits-parody_bits
  __bitstring ='0{}b'.format(totalbits-databits)

  def tobits(x):
    return [1 if digit=='1' else 0 for digit in format(x, __bitstring)[::-1]]

  table = np.zeros((np.log2(totalbits+1).astype(int),0))
  for x in range(totalbits+1):
    _ = np.array([ tobits(x) ]).T
    table = np.hstack((table.copy(), _))

  databit_pos = [x for x in range(1,totalbits+1) if not _ispowerof2(x)]

  AA = table[:,databit_pos]
  GG = np.hstack((np.identity(databits), -AA.T) )
  HH = np.hstack( (AA,np.identity(totalbits-databits)) )

  return (GG.astype(np.int),HH.astype(np.int))

class Coder(object):
  """base 4 coder decoder with string helpers.
     currently only supports 5 byte strings with 6 crc bases appended.
     coder is extending hamming code with 1 parody base over the whole payload.
     This guarentees detection of double base errors.
  """
  def __init__(self):
    self.parody_bits = 5
    (self.G,self.H) = _generate_HCMatrix(self.parody_bits)
    self.total_bits = 2**self.parody_bits-1
    self.data_bits = self.total_bits-self.parody_bits
    self.lut = np.array(["A","C","G","T"])
    self.rlut = {n:k for k,n in zip(range(len(self.lut)),self.lut)}

  def _compute_crc(self,data,size=6):
    ds = "".join(map(str,data.flatten()))
    h = sha256(ds).digest()
```

```
    base4sha = []
    for b in map(ord,h):
      base4sha = base4sha + _base4(b)

    return np.array(base4sha[-size:])


    ##### OLD algorithm
    #zeropad
    if len(data.shape) ==2:
      data = data[0]
    pad = np.mod(6-np.mod(len(data),6),6)
    data = data.copy()
    data = np.concatenate((data,[0]*pad))
    crc = np.mod(np.sum(data.reshape(-1,6),axis=0),4)
    return crc

  def string2data(self, s):
    assert len(s)==5
    data = np.zeros(5*4+6,dtype=np.int)
    bts = map(ord,s)

    ind = 0
    for b in bts:
      data[ind:ind+4] = _base4(b)
      ind +=4
    #_t = np.concatenate((data,[0,0,0,0]))
    #crc = np.mod(np.sum(_t.reshape(-1,6),axis=0),4)
    data[-6:] = self._compute_crc(data[0:(5*4)])
    return np.array([data])

  def data2string(self,d):
    """ checks and removes CRC
        returns (string, bool)
           true -> crc pass
           false -> crc fail
    """

    s = ""
    data = d[0,:20]
    crc = d[0,20:]
    computed_crc = self._compute_crc(data)
    crc_pass = np.array_equal(crc,computed_crc)
    data = data.reshape(-1,4)
    for row in data:
      d_byte = np.dot(row,[4*4*4,4*4,4,1])
      s = s+chr(d_byte)
    return (s,crc_pass)

  def data2dna(self,data):
    data = data[0]
    sdat = self.lut[data]
    return "".join(sdat)

  def dna2data(self,dna):
    return np.array([[self.rlut[k] for k in dna]])


  def secded_encode(self,data):
    cd = self.hamming_encode(data)
```

```
  cs = [[np.mod(np.sum(cd),4)]]
  return np.concatenate((cs, cd ),axis=1)

def secded_decode(self,data):
  """ returns (decoded values, error condition)
      err cases:
        -1: impossible to decode
         0: all checks out okay
         1: parody bit mismatch
         2: correctable code error
         3: correctable code error but parody still mismatches
  """
  _err = 0
  p = data[0,0]
  d = data[:,1:]
  parody_check = (p != np.mod(np.sum(d),4) )
  cd = self.hamming_correct(d)
  if isinstance(cd , type(None)):
    return (None,-1) #can't decode anything
  correction = not np.all(d == cd)
  if correction:
    parody_check = ( p != np.mod(np.sum(cd),4) )

  _err = 1*parody_check+2*correction
  return (self.hamming_decode(d),_err)


def hamming_encode(self,data):
  return np.mod(dot(data,self.G),4)

def hamming_correct(self,data):
  """doesn't remove the last parody bits"""
  G = self.G
  H = self.H
  (errpos,errval) = self._check_error(data)
  if errval == 0:
    #return data[:,0:G.shape[0]]
    return data
  elif np.isnan(errval):  #bad corruption
    return None
  else:
    fixed_data = data.copy()
    fixed_data[0,errpos] -= errval
    fixed_data = np.mod(fixed_data,4)
    return fixed_data

def hamming_decode(self,data):
  """ correct and remove parody """
  d = self.hamming_correct(data)
  if isinstance(d , type(None)):
    return d
  else:
    return d[:,0:self.G.shape[0]]

def _check_error(self,data):
  """ check for error in hamming code """
  H = self.H
  checksum = (np.mod(dot(H,data.T),4).T)[0]
  loc = (checksum!=0).astype(int)
  ind = 2**np.arange(len(loc))
```

```
    h_ind =  dot(loc,ind)
    if h_ind ==0: #cheks out!
      return (0,0)

    bitpos = np.arange(0,H.shape[1]+1)
    with np.errstate(divide='ignore'):
      nparody = np.ceil(np.log2(bitpos))
    error_bit_lut = bitpos-nparody
    for (_x,_i) in zip(2**np.arange(self.parody_bits),np.arange(self.parody_bits)):
      error_bit_lut[_x] = _i+self.data_bits+1
    #print error_bit_lut
    #print error_bit_lut[h_ind]
    err_pos = int(error_bit_lut[h_ind]-1)
    #if we're here then there's an error.

    #make sure all non-zero checksums are the same:
    err_val = checksum[checksum!=0][0]
    if np.all(err_val == checksum[checksum!=0]):
      return (err_pos,err_val)#zero indexed instead of 1 indexed
    else:
      return (np.nan,np.nan)
```

3.2 Bubble Detector

The OpenSCAD code implementing the bubble detector 3d printable part is as follows. Assembly is done by friction fitting the LED Into the round hole, inserting the light sensor into the square hole and inserting transparent 1/16 inch tube into the small through hole.

```
difference(){
  cube([10,10,20],center = true);

  cylinder(h=20,d=5,center=false,$fn=30);
  cylinder(h=40,d=1.2,center=true,$fn=30);

  translate([0,0,-3])
  rotate([0,90,0])
  cylinder(h=40,d=25.4/16+0.5,center=true,$fn=30);
  translate([-2.5,-2,-9])
  cube([5,20,3]);
}
```
